# Flexible Multimodal Wearable Biosensors for Continuous Health Monitoring: Recent Advances and Challenges

**DOI:** 10.3390/bios16080410

**Published:** 2026-07-29

**Authors:** S. Fouziya Sulthana, U. Mohammed Iqbal, B. S. Sreeja, Rajesh Anbazhagan

**Affiliations:** 1 Department of Mechatronics Engineering, College of Engineering and Technology, SRM Institute of Science and Technology, Kattankulathur, Chennai 603203, Tamil Nadu, India; fouziyas@srmist.edu.in; 2 Department of Mechanical Engineering, College of Engineering and Technology, SRM Institute of Science and Technology, Kattankulathur, Chennai 603203, Tamil Nadu, India; 3 Department of Electronics and Communication Engineering, Centre for Medical Electronics, College of Engineering, Anna University, Chennai 600025, Tamil Nadu, India; sreejaabs@annauniv.edu; 4 School of Electrical and Electronics Engineering, SASTRA University, Thanjavur 613401, Tamil Nadu, India; rajesha@ece.sastra.edu

**Keywords:** wearable, biosensors, biochemical, biophysical, multimodal sensors, continuous health monitoring

## Abstract

The integration of biochemical and biophysical sensing technologies in wearable devices represents a significant advancement in continuous and individualized health monitoring. Traditional wearable sensors are often restricted to either biophysical signals like mobility, temperature, electrophysiological activity, etc., or electrochemical analytes like glucose, lactate, etc. Integrating these complementary modalities into a single, compact, skin-conformal device creates opportunities for multimodal diagnostics and health monitoring systems. This review summarizes the recent advancements in multifunctional integrated flexible wearable biosensors that monitor chemical and physical parameters simultaneously. Initially, the insights are provided on the advances in flexible materials and fabrication techniques that support next-generation wearable devices. Subsequently, the recent progress in biophysical sensing, biochemical sensing, and strategies for their integration into multifunctional wearable platforms is critically examined. Finally, the review concludes with the key challenges to clinical translation and a perspective on future directions to achieve the next generation of integrated flexible wearable systems.

## 1. Introduction

Flexible wearable sensors have gained attention as a transformative technology in personalized health care, enabling real-time continuous health monitoring. This facilitates the early diagnosis of physiological abnormalities, real-time monitoring of chronic conditions, and preventive actions [1,2]. Traditional methods of clinical visits often miss the dynamic physiological variations, especially in conditions like cardiovascular diseases, mental health disorders, diabetes, etc., which appear with complex time-sensitive changes [3]. Early wearable technology like fitness bands and smartwatches primarily dealt with unifunctional biophysical sensing by capturing parameters like bodily temperature, pulse rate, or step count [4]. Although these devices have attracted extensive usage and established their efficacy in encouraging physical activity and basic health monitoring, their restricted sensing capabilities limit their usage in in-depth physiological and pathological investigations. They often fail to precisely address the underlying biochemical changes that are crucial indicators of metabolic, hormonal, or immunological health. Despite these limitations, standalone biophysical and biochemical wearable sensors continue to play a significant role in continuous healthcare monitoring and remain an active area of research. Biophysical wearable sensors have demonstrated excellent performance in monitoring physiological parameters such as ECG, respiration, body temperature, and body motion, providing reliable real-time physiological information for disease diagnosis and rehabilitation. Similarly, biochemical wearable sensors are capable of detecting biomarkers such as glucose, lactate, electrolytes, cortisol, and other metabolites and have shown remarkable sensitivity and specificity for personalized health monitoring. Continuous advances in flexible materials, sensing mechanisms, and wireless communication have further improved the performance, reliability, and clinical applicability of these single-modality sensing platforms. Therefore, integrated multimodal wearable biosensors extend the capabilities of standalone biophysical and biochemical sensing technologies by enabling the simultaneous acquisition of complementary physiological and biochemical information.

The multifunctional wearable biosensors provide a more detailed evaluation of the body’s physiological condition by capturing correlated signals. For example, to understand glycaemic dynamics, the sensors can combine glucose levels with physical activity data, or they can monitor lactate and muscle activity during exercise to analyze fatigue in real time. Combining biochemical sensors, which may detect perspiration, interstitial fluid, saliva, or tears, with traditional physical sensors opens new possibilities for disease diagnosis, therapy monitoring, and wellness enhancement [5,6]. Moreover, advancements in wireless communication, flexible electronics, microfluidics, and nanomaterials have facilitated the development of multimodal, skin-conformal sensor systems capable of continuously acquiring and transferring complex health data.

**Figure 1:**
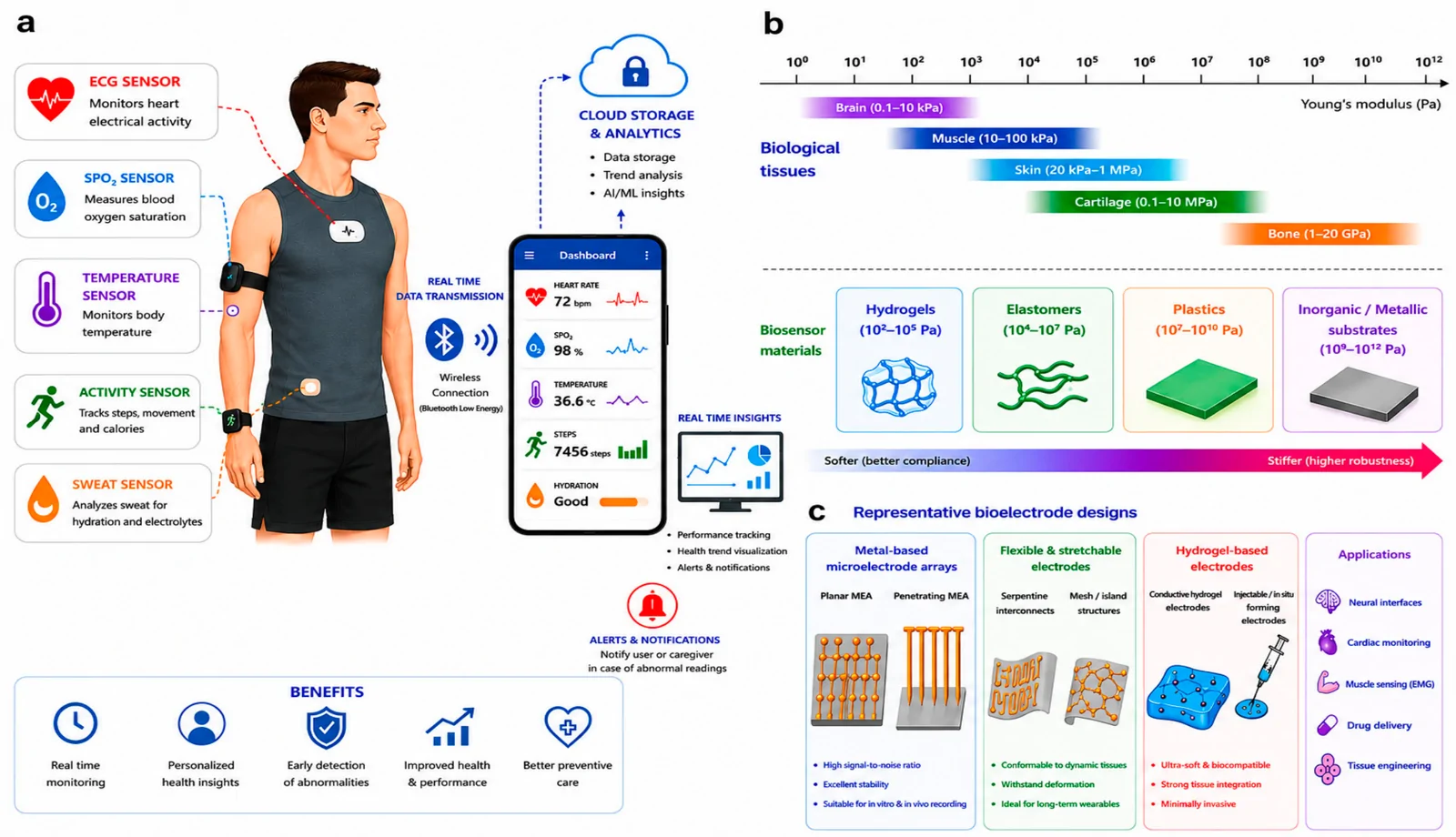
(**a**) Wearable biosensors for real-time physiological monitoring. (**b**) Mechanical properties of tissue and biosensor materials. (**c**) Bioelectrode designs.

Flexible wearable electronics are advancing rapidly, which raises the potential engineering applications for health monitoring [7]. Flexible substrates like polydimethylsiloxane (PDMS), thermoplastic polyurethane (TPU), Ecoflex, etc., enable the device to closely adhere to the body, maintaining stable contact during movement and guaranteeing user comfort for a longer duration [8]. Figure 1 highlights the substantial mechanical mismatch between conventional electronic materials and biological tissues. Because the Young’s modulus of silicon and metallic conductors is several orders of magnitude greater than that of skin, direct integration can lead to poor conformal contact, discomfort, and motion-induced artefacts. Consequently, flexible substrate materials and stretchable interconnects have been developed to improve mechanical compatibility while maintaining stable electrical performance during body movements.

Several recent reviews have highlighted the remarkable potential of wearable biosensors, which address aspects including real-time monitoring, multi-metabolite detection, and non-invasive sensing [9,10,11,12]. However, this paper discusses the detection targets, fabrication methods, and multimodality of integrated biosensors, which have been rarely addressed in earlier works. The main objective of this paper is to provide a comprehensive survey of the recent research carried out in the field of biophysical and biochemical integrated flexible wearable sensors, emphasizing the technology, methods, and applications over the past decade. Primary attention has been placed on the design techniques for multimodal integration, challenges faced in real-world deployment, and the future directions of this domain.

## 2. Flexible Materials and Fabrication Techniques

The design and development of multifunctional wearable biosensors that combine biochemical and biophysical modalities significantly rely on the selection of appropriate materials and their fabrication to achieve a balance between biocompatibility and long-term stability. These materials are designed to mimic the human skin, which enables them to adhere to tissue surfaces. The flexible substrates offer mechanical support to the biosensors; the stretchable conductors enable the transmission of signals, and the active layer facilitates the sensing mechanism, which are the main components of flexible biosensors. The main categories of materials that are used for the fabrication of flexible sensors are carbon-based materials, thin metal films, and flexible polymers.

### 2.1. Materials for Flexible Substrates

Flexible substrates are the fundamental layer of wearable sensors, which determines the stretchability, mechanical compliance, and user comfort during long-term usage. Materials like polymers, silicones, and rubbers are often chosen as substrates because of their flexibility and adaptability. Among them, polydimethylsiloxane (PDMS), a commercially available silicone elastomer, is particularly popular due to its remarkable properties. It offers extremely high stretchability (up to 1000%) and is easy to process [13]. Ecoflex^®^ rubber (Smooth-On, Inc., Macungie, PA, USA) is a recently developed silicone material known for being highly stretchable and skin-friendly. With its lower modulus and superior elasticity, it is particularly suitable for sensors that demand extreme flexibility and stretchability [14].

On the other hand, polyethylene terephthalate (PET), despite its relatively high modulus (around 2–4 GPa), which limits its stretchability, is valued for its excellent transparency (>85%), strong creep resistance, and good printability. These features make PET a common choice as a substrate film in wearable electrochemical sensors [15,16]. Another widely used option is polyimide (PI) film, which stands out for its ability to retain flexibility, tensile strength, and resistance to creep even under harsh conditions such as high temperatures (up to 360 °C) and exposure to acids or alkalis.

Thermoplastic polyurethane (TPU) is a highly adaptable polymer that exhibits combined abrasion resistance and notable mechanical flexibility. Unlike PDMS, TPU is thermoplastic, making it simple to process with techniques like 3D printing and roll-to-roll manufacturing. This makes it especially attractive for extensive production of wearable patches, where performance and scalability are crucial. Table 1 gives a comparison of common flexible substrates for wearable sensors.

The mechanical compatibility between wearable devices and the biological tissue to which they are attached is a critical design consideration. Human skin typically exhibits an effective Young’s modulus ranging from approximately 20 kPa to 1 MPa depending on anatomical location, hydration level, age, and measurement conditions. In contrast, mechanically stressed areas like the foot’s sole demonstrate significantly greater effective stiffness owing to the dense keratinized tissue. Consequently, the selection of substrate materials should not rely solely on their mechanical properties but also on the intended wear location and application. Soft elastomers such as Ecoflex and PDMS are preferred for epidermal patches because their low modulus allows intimate skin conformability and minimizes motion-induced artefacts. In contrast, TPU is more suitable for smart textiles and wearable garments because of its excellent abrasion resistance and mechanical robustness. Materials such as PET and polyimide are generally selected for applications requiring dimensional stability and precise electronic integration, including flexible electronic circuits, contact lens electronics, and hybrid sensor platforms.

### 2.2. Materials for Sensing Layer

The sensing layer is the crucial part of the wearable sensor, which responds to external stimuli and converts them into measurable signals. The materials utilized for this layer predominantly determine the sensor’s sensitivity, reliability, and efficacy in practical usage. To improve performance, the researchers are experimenting with various materials, including carbon-based materials, nanostructured metals, and conductive polymers for developing effective sensing layers.

#### 2.2.1. Carbon-Based Materials

Carbon-based materials are utilized in flexible sensors due to their exceptional conductivity, flexibility, and stability. These materials include graphene, Carbon Nanotubes (CNT), reduced Graphene Oxide (rGO), and emerging carbon nanostructures [17]. They are lightweight in nature and are increasingly used in flexible electronics. These materials are frequently combined with polymers to create highly conductive composites [18].

Graphene is widely recognized for its excellent electrical conductivity and exceptionally large surface area relative to its mass. Despite these advantages, producing graphene on a practical scale remains challenging. To overcome this, researchers often turn to graphene oxide, a closely related derivative that contains oxygen-bearing functional groups. Unlike pure graphene, graphene oxide disperses well in water, exhibits viscoelastic behavior, and offers high viscosity along with good printability, making it more suitable for sensor fabrication.

CNTs are one-dimensional carbon structures that are known for their superior electrical conductivity and mechanical strength [19]. A single CNT can demonstrate extremely high strain sensitivity, with a gauge factor over 1000, but fabricating devices from individual CNTs is difficult and impractical for large-scale applications. To overcome this, CNT powders are usually blended with polymer substrates, where their superior conductivity significantly contributes to the development and performance of wearable sensors.

Carbon nanotubes (CNTs) and MXenes mutually enhance the performance of carbon-based materials due to their shared features, including excellent biocompatibility, intrinsic degradability, and similar electrical conductivity. MXenes are particularly useful in biomedical applications because of their hydrophilic nature, high diffusivity, and the existence of adjustable surface functional groups [20,21], which enable them to produce unique biological effects.

Beyond basic electrical conductivity and mechanical elasticity, the sensing efficacy of carbon-based materials depends heavily on quantitative biosensing parameters, including strain sensitivity, limit of detection, signal drift, and resistance to sweat matrix interference. For mechanical strain sensing, individual CNTs demonstrate extreme strain sensitivity (gauge factor > 1000), whereas CNT-polymer composites typically achieve gauge factor ≈2–50 with linear response up to 100% strain. However, carbon-based nanomaterials often suffer from non-specific biofouling in sweat due to hydrophobic interactions, leading to baseline signal drift over extended monitoring. To overcome these limitations, multi-component hydrogel matrices such as polyvinyl alcohol (PVA)/silk fibroin/graphene nanocomposites have been engineered to achieve self-healing, ultra-stretchability, and eco-friendly properties while stabilizing the electrical interface against sweat interference. Furthermore, hybrid architectures combining CNTs and MXenes leverage high diffusivity and hydrophilic surface functional groups to suppress non-specific protein adsorption, thereby lowering the electrochemical detection limit for biochemical analytes while maintaining high signal-to-noise ratios in perspiration.

#### 2.2.2. Thin Metal Films

Metals are frequently used in wearable sensor design because of their superior electrical conductivity. The commonly used metals include gold (Au), silver (Ag), platinum (Pt), and copper (Cu), which can be fabricated into ultrathin layers or nanostructures [22]. When compared with carbon-based and polymer-based sensing materials, metals are primarily used in conductive pathways. These materials maintain their ability to conduct electricity even when subjected to repeated mechanical deformation. Gold thin films are frequently used in wearable biosensors because they are easily modified with biomolecules and they are chemically stable. These sensors are safe for contact with the human body, which enables the reliable detection of health-related substances like glucose, lactate, and cortisol in sweat or interstitial fluids [23].

Silver thin films, particularly silver nanowire (AgNW) networks, are frequently employed in wearable strain and pressure sensors due to their optical transparency and their ability to conduct electricity efficiently. They create stretchable electrodes that can adapt to the body’s bending and stretching without compromising functionality when embedded in soft materials like PDMS [24]. Platinum thin films are widely utilized in wearable sensors due to their strong catalytic activity, which makes them suitable for electrochemical sensing applications. Platinum films can operate consistently in biofluids without significant degradation over time. They are frequently applied on flexible substrates in the form of ultra-thin layers, which enable the sensor to bend and conform to the skin, making it suitable for wearable health monitoring systems [25]. Additionally, copper thin films are becoming more popular as an affordable substitute, albeit issues like oxidation must be resolved with hybrid structures or protective coatings [26,27].

While thin metal films (e.g., Au, AgNWs, Pt) offer low sheet resistance (<10 Ω/sq), their application in wearable biosensing requires careful decoupling of mechanical strain from electrochemical signals. For biophysical strain sensing, biomimetic structures patterned on polydimethylsiloxane (PDMS) substrates such as micro-crack arrays or lotus-leaf hierarchical structures enable ultra-high linearity (R^2^ > 0.99) and broad working ranges without catastrophic conductive path failure. For biochemical sensing, noble metal films like gold provide low detection limits (LOD < 1 μm) for analytes like glucose and lactate due to fast heterogeneous electron transfer rates. Nevertheless, metallic films are vulnerable to sweat chloride ion (Cl^−^) corrosion and baseline drift during body movement, requiring protective encapsulation or permselective outer membranes (e.g., Nafion or polyurethane) to block interfering species such as ascorbic acid and uric acid [28,29].

#### 2.2.3. Flexible Polymers

Polymer materials have been extensively explored as active layers in flexible and wearable sensors. This is due to their favorable electrical, electromechanical, or electrochemical capabilities and their mechanical compatibility with the soft substrates employed in wearable systems. One of the key benefits of polymer sensing materials is that the elastic modulus of these materials is often close to that of the flexible substrate used, which in turn improves the durability [30]. Many active sensing layers have been made of conductive polymers, like poly(3,4-ethylenedioxythiophene) (PEDOT) and its derivative PEDOT: PSS, because of their excellent thermal stability, optical transparency, and adjustable electrical conductivity. PEDOT: PSS is especially interesting due to its compatibility with low-cost printing techniques like screen printing, inkjet printing, dip coating, and spin coating. However, the isolated PEDOT: PSS films may suffer from being fragile and decreasing conductivity due to the rigid domains under the polymer matrix. To overcome these constraints, it is usually enclosed between the flexible layers or impregnated into porous substrates, thereby improving mechanical stability [31,32].

Another commonly used polymer for wearable sensors is polyvinylidene fluoride (PVDF) and its copolymer PVDF-TrFE. These materials can generate electrical signals when they are bent or pressed, which makes them very useful for sensing motion and pressure. Their flexibility in the sensing layers allows them to follow body movements without discomfort. PVDF-based sensors are extensively utilized to measure parameters including heart rate, respiratory patterns, walking motions, and foot pressure. Their good sensitivity and stable performance over time make them reliable for long-term health monitoring [33,34]. Other conducting polymers such as polypyrrole (PPy), polyaniline (PANI), and poly(3-hexylthiophene) (P3HT) are also utilized in wearable sensors. These polymers are suitable for strain, pressure, and chemical sensing applications because they can react to chemical or mechanical deformation. Their adaptability enables integration into wearable, soft devices that can closely track bodily motions. Therefore, they are helpful in the development of comfortable health monitoring systems [35,36,37].

Conductive polymers such as PEDOT: PSS and PANI offer intrinsically soft interfaces matching human skin compliance. When configured as organic electrochemical transistors (OECTs), PEDOT: PSS exhibits significant signal amplification, yielding sub-micromolar detection limits for biofluids. However, conductive polymer films exhibit hygroscopic swelling when exposed to sweat, leading to noticeable baseline drift and impedance variations over time. To stabilize performance, crosslinkers (e.g., silane coupling agents) and self-decoupling designs are employed to isolate pressure/strain, proximity, and temperature stimuli, ensuring low signal crosstalk in composite sensing environments. Table 2 gives a comparison of the sensing performance of representative flexible sensing materials.

### 2.3. Fabrication Techniques

**Figure 2:**
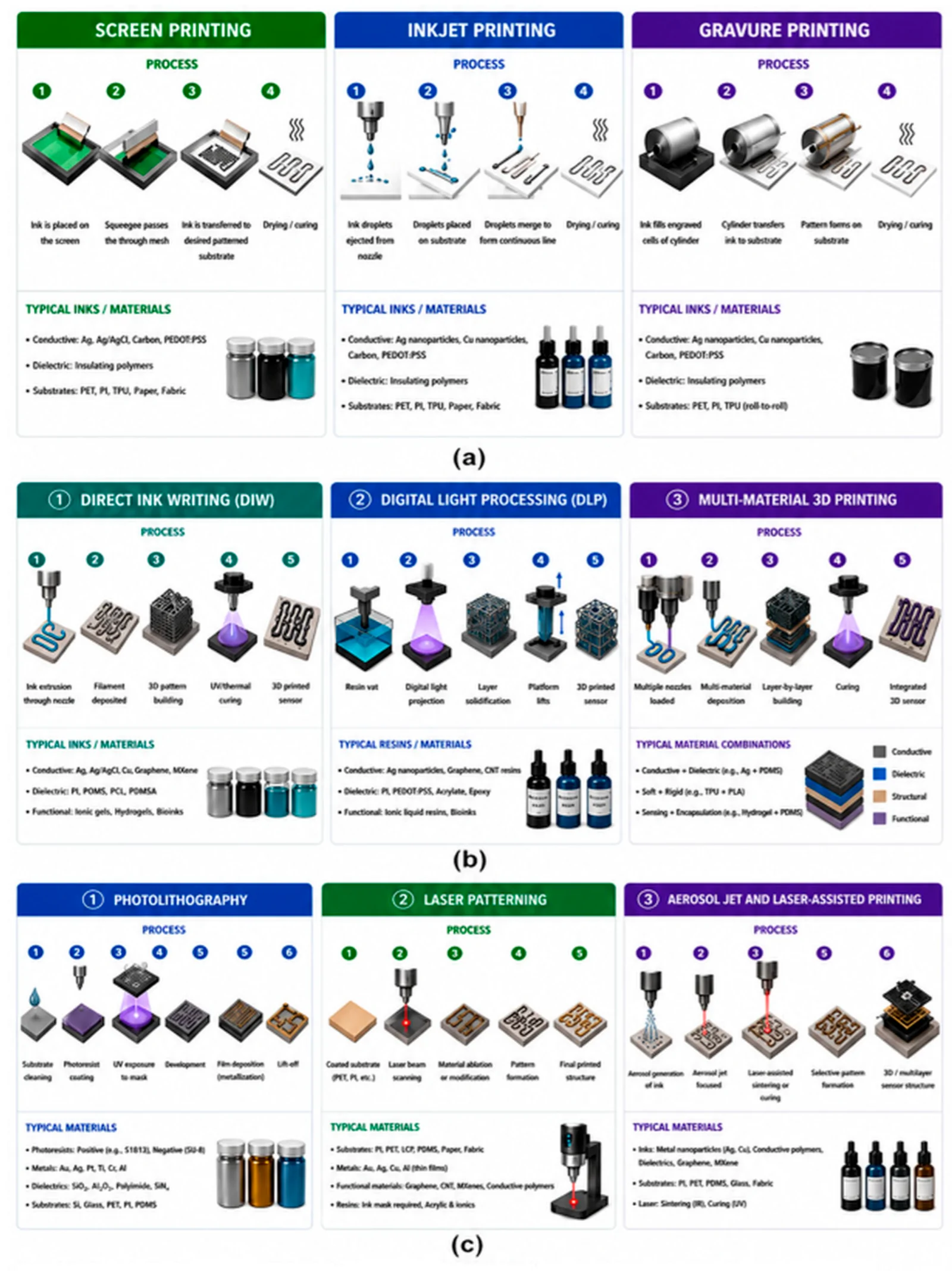
Fabrication process and materials involved in (**a**) Printed electronics, (**b**) Direct-Write 3D Printing, and (**c**) Laser and Lithographic Printing. Note: Ag = Silver, AGCL = Silver Chloride, CNT = Carbon Nanotube, CNC = Cellulose Nanocrystal, MXene = Transition Metal Carbide/Nitride, PEDOT: PSS = Poly(3,4-ethylenedioxythiophene): Polystyrene Sulfonate PET = Polyethylene Terephthalate, PI = Polyimide, PDMS = Polydimethylsiloxane, PCL = Polycaprolactone, TPU = Thermoplastic Polyurethane, LCP = Liquid Crystal Polymer.

Fabrication techniques have a significant impact on the accuracy and precision of measurements and impose a variety of constraints on fabrication time and cost. Many fabrication techniques for wearable devices are reported in the literature [38]. Flexible and wearable sensors are mainly fabricated using printed electronics, direct-write 3D printing, laser/lithographic printing, etc. Each method offers a trade-off among scalability, material choices, durability, and environmental stability. The process carried out in the fabrication and the material used are illustrated in Figure 2.

#### 2.3.1. Printed Electronics

This technology involves the fabrication of electronic components and circuits by depositing functional inks onto flexible or rigid substrates. It utilizes the cost-effective techniques such as screen printing, inkjet printing, gravure printing, etc., to create components. Wearable sensors can be printed with or without the use of masks, depending on the implementation method.

Screen printing is a common printing method that requires a mask. A fill blade or squeezer is used to push functional ink into screen nozzles, where it is then applied to the substrate. Upon the removal of the mask, functional ink will persist on the substrate, creating a patterned film. The screen-printed wearable sensors are extensively developed for pressure monitoring, strain sensing, sweat analysis, etc. It is also widely used for electrocardiography (ECG), electromyography (EMG) [39,40].

Inkjet printing is a non-contact additive manufacturing method that precisely deposits conductive and functional inks onto flexible substrates. This technique has been widely adopted in sensor development. The functional inks usually consist of a silver nanoparticle, graphene, carbon nanotubes (CNTs), MXenes, or conductive polymers like PEDOT: PSS. It should overcome the limitations on solubility, viscosity, surface tension, etc., which can increase the process cost to a certain extent. Inkjet-printed sensors have been effectively utilized for the monitoring of strain, pressure, temperature, glucose, and electrophysiological signals [41,42,43].

Gravure printing is an impact-based printing technique that produces high-quality prints quickly and affordably. It makes use of low-viscosity inks that are applied to the substrate using a gravure cylinder under high pressure. The impression cylinder, usually made of rubber, controls the movement of the substrate, and the gravure cylinder, usually made of copper-coated steel, is used for the transmission of inks. The gravure printing technology is used in the development of numerous electrochemical sensors [44,45].

#### 2.3.2. Direct-Write 3D Printing

Direct-write (DW) is an additive manufacturing method that directly transfers material onto the substrate and enables the fabrication of wearable sensors with complex 3D geometries and adjustable mechanical and electrical properties. It is a flexible fabrication technique that enables precise material deposition on substrates without the use of molds or masks. A variety of functional inks can be patterned into two-dimensional and three-dimensional architectures using extrusion-based DW assembly, with feature resolutions as fine as 250 nm. This approach uses compressed air to extrude inks with precisely regulated rheological characteristics through a nozzle that ranges in diameter from 1 to 500 μm. A key benefit of DW assembly is its compatibility with many ink systems, including colloidal suspensions, hydrogels, polymer melts, sol–gel materials, and nanoparticle-infused inks.

Direct ink writing (DIW), a subset of DW, allows the fabrication of three-dimensional structures with distinctive geometries. In the DIW process, conductive nanomaterials, including carbon nanotubes, graphene, silver nanowires, and MXenes, are suspended in viscoelastic inks and precisely deposited by an extrusion-based printing mechanism to create functioning electronic devices [46,47].

Digital Light Processing (DLP) is a three-dimensional printing method that uses light to polymerize photosensitive resin gradually. This technique may generate a sensor architecture with high accuracy and complex geometries. Recent developments in DLP-printed organohydrogels have resulted in the creation of biocompatible, extremely stretchy, and self-adhesive wearable sensors. These sensors are capable of efficiently tracking pressure-related physiological signals as well as human movements. DLP offers faster fabrication speeds and better spatial resolution than extrusion-based printing methods [48,49].

Multi-material printing enables the simultaneous fabrication of various materials in a single manufacturing process. Conductive, dielectric, and structural materials can be simultaneously deposited to fabricate fully integrated wearable devices. This facilitates the construction of sensors, electrical interconnects, and protective encapsulating layers within a singular structure [50,51].

#### 2.3.3. Laser and Lithographic Printing

Laser-based and lithographic fabrication methods provide high-resolution patterning capabilities required for advanced wearable sensors and bioelectronics. For the creation of microscale sensor structures, photolithography is still one of the most well-established methods. Patterns from a photomask are transferred onto a substrate coated with photoresist by means of ultraviolet exposure. Photolithography has been widely used in the fabrication of flexible electrodes, microfluidic biosensors, and stretchable electronic circuits.

Laser-based fabrication is a fast and accurate method for generating patterns on sensor materials without the requirement of masks. Techniques like laser-induced graphene (LIG), laser ablation, and laser engraving are extensively employed in the production of wearable sensors. These approaches can directly create conductive microstructures on polymer substrates, removing any need for complex chemical etching procedures. Laser-reduced GO has been used as a sensing element in numerous wearable sensors and has a lot of potential for high-performance artificial skin and throats [52,53].

Emerging methods like laser-assisted printing and aerosol jet printing integrate the benefits of high-resolution patterning with additive manufacturing. These techniques can accurately create complex microscale structures and extremely fine electrical traces. Additionally, they make gradient interfaces possible, which improve the stretchability of wearable devices and minimize the movement-related signal interruptions. Due to this, wearable biosensors can be made more reliable and perform better [54,55].

## 3. Biophysical Sensing in Wearables

A variety of sensors have been developed in recent years for monitoring various physiological signals. Many studies mentioned that the different configurations of flexible sensors, such as piezoresistive sensors, piezoelectric mechanical devices, and capacitive sensors, exhibit high sensitivity for monitoring human physiological signals [56,57,58]. These sensors monitor physiological signals related to the cardiovascular, respiratory, neurological, musculoskeletal, and thermoregulatory processes. The accuracy and functionality of wearable biophysical sensors have been greatly enhanced by the combination of flexible electronics with advanced signal processing techniques.

### 3.1. Cardiovascular Monitoring

Cardiovascular diseases are still the biggest cause of death worldwide, which is why researchers have been putting so much effort into wearable technologies that can track heart health continuously rather than relying on occasional hospital visits. One of the most widely used approaches is the flexible electrocardiogram (ECG) sensor, which provides real-time measurement of cardiac electrical activity through skin-mounted electrodes without causing much discomfort. The skin-contactable ECG sensor allows users to easily identify their cardiac condition and diagnose critical cardiac problems early, such as cardiomyopathy, arrhythmia, and high blood pressure [59,60]. Recent advancements in dry electrodes, conductive hydrogels, graphene-based electrodes, and textile-integrated ECG systems have significantly enhanced signal quality and long-term wearability. Conventional physiological monitoring systems typically rely on gel-based Ag/AgCl electrodes. However, their long-term use is often associated with several limitations, including skin irritation and discomfort [61,62]. To address these issues, researchers have increasingly focused on flexible dry electrodes because of their soft, skin-conformal, and non-invasive properties.

A flexible wearable electrode designed for ECG monitoring has been reported in [63]. In this study, a highly conductive silver layer was deposited onto a cowhide-based flexible substrate using plasma sputtering technology. ECG measurements were conducted on six participants, and the results showed that the signal quality obtained from the proposed electrode was comparable to that of standard Ag/AgCl electrodes. An additional advantage of the cowhide substrate is its compatibility with textile manufacturing, making it suitable for the development of garments integrated with flexible sensing systems. This feature may also enhance user acceptance and comfort during long-term wear. Flexible ECG patches can continuously monitor arrhythmias, atrial fibrillation, myocardial ischemia, and heart rate variability in non-clinical environments [64].

Photoplethysmography (PPG) has become another dominant modality for cardiovascular monitoring. Flexible PPG sensors measure blood volume changes using optical methods and are extensively integrated into smartwatches and epidermal patches. Combined ECG–PPG systems enable cuffless blood pressure estimation through pulse transit time analysis, representing a major advancement toward continuous cardiovascular assessment [65,66]. Recent advances in flexible wearable technologies have led to the development of highly sensitive pulse-monitoring sensors that can be comfortably attached to the skin. One notable example is a patch-based strain sensor [67] utilizing polyaniline as the sensing material, which combines excellent flexibility with high sensitivity to detect pressure fluctuations generated by blood flow. The recorded pulse signals can be analyzed to extract clinically relevant cardiovascular information.

Further improvements have resulted in flexible pressure sensor arrays [68] capable of simultaneously monitoring pulse signals at three different locations. These systems employ ionogel-based sensing arrays fabricated on flexible PET substrates, where arterial pressure variations are converted into measurable resistance changes. In another approach, sensors based on poly (vinylidene fluoride-co-trifluoroethylene) (PVDF-TrFE) integrate sensing elements with analog signal amplification circuitry on a flexible platform. These devices can detect low-pressure stimuli and significantly enhance weak electrical signals, making them highly effective for continuous and accurate pulse-rate monitoring [69]. Lee et al. designed a disposable, skin-conformal wireless wearable cardiac sensor with flexible electronics and soft bioadhesive layers to achieve intimate skin contact and reliable ECG acquisition. The device incorporates wireless power harvesting and water-resistant packaging, enabling continuous monitoring during daily activities. Clinical studies in atrial fibrillation patients confirmed performance comparable to conventional Holter monitors with improved user comfort [70].

### 3.2. Respiratory Monitoring

The study of various parameters of respiration gives us valuable information on lung function, metabolism, sleep, cardiovascular function, and infection. The COVID-19 pandemic has led to increased interest in continuous monitoring of respiratory parameters beyond typical clinical settings. Recently, flexible wearable respiratory sensors have attracted great interest.

The respiratory sensors currently used in wearables rely on various sensing methods, including but not limited to strain sensing, piezoresistive sensing, capacitive sensing, triboelectric sensing, piezoelectric sensing, and bioimpedance analysis. Strain sensors placed on the chest, made from conductive elastomers, carbon nanotubes, graphene, or MXene composites, can effectively track thoracic expansion and contraction during respiration [71]. The mechanical deformation is converted into an electrical signal, which is correlated with the respiratory rate, tidal volume, and breathing patterns. However, continuous contact with the skin may induce irritation.

Fiber Bragg grating (FBG) sensors are frequently utilized in respiratory rate monitoring applications because of their ease of use, stretchability, compact size, and resistance to electromagnetic interference [72,73]. Two main methods are typically used to monitor respiratory rate using FBG sensors. In the first approach, the sensor measures the expansion and contraction of the chest, which results in variations in chest volume throughout the breathing cycle, in order to identify respiratory activity. In the second approach, the sensor detects changes in temperature and humidity linked to intake and exhalation to determine the respiratory rate [74].

A wearable respiratory monitoring system for workers operating in high-pressure environments was developed by integrating FBG sensors into garments, thereby creating a smart clothing platform [75]. In this design, the optical fiber was coated with polyimide (PI), and the FBG was embedded within a flexible matrix to enhance sensor durability, sensitivity, and adaptability to individuals with different body dimensions. The sensing mechanism relies on translating the mechanical deformations caused by breathing into measurable wavelength shifts in the FBG. Using a similar approach, another study incorporated five FBG sensors into an elastic textile band to continuously monitor respiratory rate by detecting breathing-induced strain variations [76].

### 3.3. Body Temperature Monitoring

Body Temperature is a simple physiological parameter but a powerful marker for infection, inflammation, metabolic disorder, disturbed circadian rhythm, and overall physiological state. Thermometers measure temperature with isolated values that do not represent the fluctuation that occurs continuously all day long. Temperature sensors in wearable devices are flexible and enable monitoring of temperature in real-time under practical conditions.

Many kinds of flexible temperature sensors have been proposed; however, the potential of flexible wearable devices to continually provide energy is essential for practical use. Chen et al. developed a flexible temperature sensor system incorporating energy harvesting, monitoring, and reporting systems [77]. The system is built with temperature sensor arrays and a triboelectric generator. In addition, they are made on a pliable PDMS substrate for monitoring minute temperature changes. In the system, a triboelectric nanogenerator is utilized as an energy source. To deal with the high internal impedance of the triboelectric nanogenerator, a power management circuit is used. The circuitry guarantees the successful delivery of energy and ensures the stable functioning of the temperature sensing element.

A flexible and wireless temperature sensing system was created utilizing PEDOT: PSS as the sensing material in [78]. The sensor was covered with a fluorinated polymer to mitigate the impact of humidity on temperature data. Here, the flexible electrical circuit was produced by inkjet-printing silver ink onto a polyethylene naphthalate (PEN) substrate. The system was capable of measuring body temperature in real time, interpreting the data, and wirelessly transferring the readings to a smartphone for remote monitoring. Khan et al. developed a wearable sensor patch to continuously monitor ECG signals and body temperature [79]. The temperature sensor uses a thermistor composed of nickel oxide nanoparticles embedded in a polystyrene-butadiene binder. Temperature variations resulted in variations in the thermistor’s resistance, which were quantified using a voltage divider circuit. The temperature data were subsequently digitized and wirelessly transmitted to a host computer via Bluetooth for further analysis and processing.

In contrast to traditional wireless sensing systems that depend on batteries, a flexible, battery-free temperature sensing platform that is powered and operated with Near Field Communication (NFC) was proposed in [80]. The system was developed to evaluate skin conditions, including blood perfusion, hydration levels, and tissue injuries, by tracking variations in the thermal conductivity of the skin through a Cr/Au-based temperature sensor. The device incorporated an inductive coil, a microprocessor, and additional passive electronic components to harvest energy from radio-frequency (RF) signals while enabling wireless data transmission.

Colorimetric temperature sensors often provide lower temperature resolution and precision when compared to resistive temperature sensors and thermistors, which makes them less suitable for continuous long-term monitoring. Flexible resistive temperature sensors and thermistors, on the other hand, offer several benefits, such as small size, straightforward circuit design, and simple skin integration. These advantages enabled their utilization in various healthcare technologies, including the estimation of core body temperature [81], enhancement of temperature-sensitive biosensor accuracy via calibration [82], and mitigation of skin ulcer risk through continuous skin temperature monitoring [83].

### 3.4. Motion and Activity Monitoring

Human motion analysis is considered one of the most developed applications of wearable sensing technologies. Monitoring movement patterns continuously provides significant information on physical activity, progress of rehabilitation, musculoskeletal conditions, neurological disorders, and performance in athletic activities. Continuous monitoring of body movements has become increasingly important in healthcare because it allows clinicians to detect abnormalities at an early stage and intervene before conditions worsen. Many neurological and metabolic disorders manifest through changes in movement patterns. For example, abnormal posture, impaired gait, and involuntary hand tremors are commonly observed in patients with diseases such as diabetes, Alzheimer’s disease, and Parkinson’s disease [84,85,86]. Detecting these subtle changes early can significantly improve disease management and treatment outcomes.

Wearable medical devices have emerged as effective tools for tracking body movements in real time. Unlike conventional clinical assessments that provide only periodic observations, wearable sensors can continuously record motion throughout daily activities. This capability makes it possible to identify sudden tremors, irregular movements, and other symptoms that may otherwise go unnoticed. As a result, the development of wearable sensors for continuous motion monitoring is gaining considerable attention for early diagnosis, long-term patient monitoring, and personalized healthcare interventions [87].

Normal and coordinated movement of the hands and limbs is essential for performing routine tasks, maintaining productivity, and ensuring a good quality of life. Even small changes in hand movement can provide valuable insights into an individual’s physical and neurological health. The feet are another important part of the body for movement assessment because they support most of the body’s weight during standing, walking, and running. This is especially relevant for athletes and manual workers who are exposed to repeated mechanical loading. Continuous monitoring of pressure distribution across the soles of the feet can help identify abnormal loading patterns, detect gait-related issues, and reduce the likelihood of injuries [88,89]. Such information is valuable for both preventive healthcare and performance optimization.

Strain sensors developed by integrating metallic conductive components within an elastomeric substrate have been used to assess the bending angles of primary joints, including the hip, knee, and ankle. During joint flexion, tensile strain develops on the outer surface of the joint while compressive deformation occurs on the inner surface. Flexible strain sensors attached to the skin experience corresponding deformation, producing proportional resistance or capacitance changes that can be correlated with joint angle and movement velocity. Consequently, sensor placement over regions of maximum skin deformation is essential for achieving high measurement sensitivity and repeatability. Di Tocco et al. effectively monitored the knee joint movements by directly integrating a flexible strain sensor into textile materials [90]. The sensor components were enclosed to improve measurement reliability by reducing errors caused by external conditions and mechanical disturbances. The sensors were manufactured in various sizes and shapes, allowing for adaptability to different body types and ensuring comfortable long-term wear.

**Figure 3:**
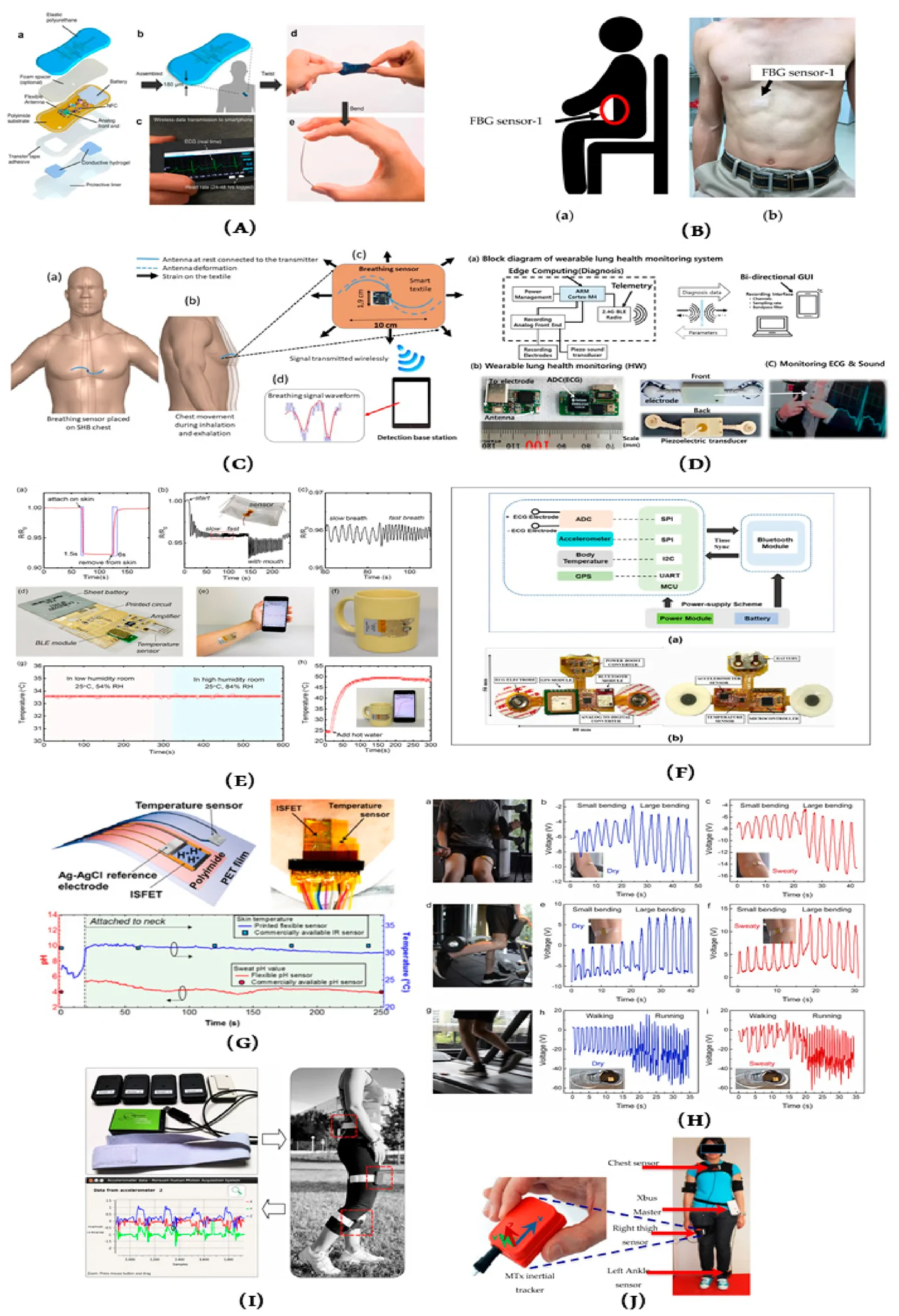
Examples of biophysical wearable sensors (**A**) Flexible cardiac sensor in a thin elastic enclosure. (**a**) Exploded view of wearable cardiac sensor (**b**) assembled sensor device (**c**) wireless data transmission to smartphone (**d**,**e**) sensor in mechanical twist and deformation [70] (**B**) FBG sensor for respiratory monitoring. (**a**) subject’s posture (**b**) FBG sensor installation position [93] (**C**) Schematic representation of the breathing sensor. (**a**) Sensor placed on the human body (**b**) sensor is stitched onto the elastic textile, and it follows the movement of the chest wall (**c**) sensor strained by chest movement, so the transmitted signal changes during breathing (**d**) Signal detected wirelessly [94] (**D**) Wireless wearable respiratory monitoring system [95] (**E**) Wearable body temperature monitoring sensor. (**a**) Resistance response of sensor with human skin (**b**) breathing rate and breathing type monitoring (**c**) Enlarged image of data (**d**) Optical image of wireless temperature sensing platform with printed temperature sensor (**e**,**f**) Schematic of wireless sensing platform on arm and coffee cup (**g**,**h**) Real-time temperature monitoring of skin and cup [78] (**F**) Flexible biosensor patch for physiological monitoring. (**a**) Operation scheme of the proposed device (**b**) prototype of biosensor [96] (**G**) Wearable flexible sensor with pH and temperature monitoring [97] (**H**) Sweat-resistant triboelectric sensor. (**a**) Photo of the sensor attached on the elbow joint for dumbbell biceps curl monitoring. dumbbell biceps curl monitoring results (**b**) before and (**c**) after sweating. (**d**) Photo of the sensor attached on the knee joint for leg curl monitoring. Leg curl monitoring results (**e**) before and (**f**) after sweating. (**g**) Photo of the sensor attached on the sole for running monitoring. Running monitoring results (**h**) before and (**i**) after sweating [98] (**I**) Proposed system architecture for human motion monitoring using accelerometer [99] (**J**) Inertial tracker and sensor placement [100]. Reprinted with permission from corresponding references.

Recent developments in flexible inertial measurement units (IMUs) have significantly improved the potential to measure human movement in a comfortable way [91,92]. These systems include accelerometers, gyroscopes, and magnetometers on flexible or elastic substrates, enabling them to adhere to the body’s surface. Flexible IMUs can precisely measure body orientation, acceleration, gait patterns, and overall movement dynamics by combining data from these sensors. They are useful in a variety of healthcare applications, such as rehabilitation monitoring, fall detection, neurological condition assessment, and human activity recognition, due to their ability to continuously collect detailed motion information. Although each of the sensing modalities discussed in this section provides valuable physiological information independently, they also serve as the fundamental sensing components for integrated multimodal wearable biosensing systems. The complementary integration of these biophysical sensing modalities with biochemical sensing technologies enables more comprehensive assessment of human health, which is discussed in Section 5. Table 3 summarizes the major biosensing physical modalities in wearable health monitoring. Figure 3 gives examples of wearable biophysical sensors proposed in the literature in recent years.

## 4. Biochemical Sensing in Wearables

Biophysical sensors provide essential physiological and biological information, whereas biochemical sensors provide direct measurement of molecular indicators relevant to metabolism, hydration, stress, disease progression, and treatment response. The continuous real-time monitoring of biomarkers is a significant advancement in wearable healthcare technologies. Wearable biochemical sensors are designed to detect biomarkers present in various biofluids, including human sweat, saliva, tears, interstitial fluid (ISF), and wound exudate. Biofluid information can be passively obtained by a non-invasive or minimally invasive method. Recent advancements in electrochemical sensors, microfluidics, innovative nanomaterials, and flexible electronics have significantly enhanced the development of multifunctional wearable biochemical devices.

### 4.1. Sweat-Based Biosensing

Sweat has been considered the most extensively researched and analyzed biofluid for wearable biochemical sensing because of its wide availability and significant biomarker composition. Sweat comprises several analytes such as glucose, lactate, sodium, potassium, chloride, cortisol, uric acid, and inflammatory indicators [101]. Accurate sweat collection is essential for reliable real-time biochemical sensing because sweat production varies significantly between individuals and across different body regions. Factors such as sweat rate, pH, salinity, sample volume, and analyte degradation can influence measurement accuracy, making it challenging to correlate sweat biomarkers with physiological conditions [102].

Conventional techniques used for collecting sweat are whole-body washdown, absorbent patches, and Macroduct^®^ devices (ELITechGroup Inc., Logan, UT, USA) [103]. However, they often require controlled laboratory environments, trained personnel, and sufficient sweat volumes in addition to challenges like sample evaporation, contamination, regional variability, and delayed analysis. To address these limitations, researchers have developed advanced wearable sweat-sampling technologies that integrate sweat collection and sensing within a single platform. These include engineered skin sensor interfaces that minimize contamination, absorbent materials that improve comfort and sweat uptake, sweat-guiding structures that direct sweat toward sensing regions, and microfluidic systems that transport sweat through miniature channels using capillary and glandular pressures [104,105].

In recent years, considerable progress has been made in the development of flexible and wearable sweat sensors for continuous health monitoring. However, the dynamic and complex characteristics of sweat production necessitate the real-time and multiplexed monitoring of target biomarkers. Therefore, an integrated wearable sensor array was proposed by Gao et al. to enable in situ analysis of sweat and provide comprehensive physiological information [106]. This wearable skin patch combines several high-performance electrochemical sensors with a flexible printed circuit board (FPCB) to process signals and transmit data wirelessly. The device can simultaneously detect important sweat biomarkers, including glucose, lactate, sodium (Na^+^), and potassium (K^+^), while also monitoring skin temperature. The wearable system effectively monitored changes in clinically relevant analytes during prolonged exercise. Its functionality was later extended to detect a broader range of biomarkers, such as pH, calcium (Ca^2+^), chloride (Cl^−^), heavy metals, and other chemical substances [107,108,109].

Jia et al. developed the lactate sensor with a composite of carbon nanotubes (CNTs), tetrathiafulvalene, and chitosan applied to a screen-printed carbon electrode [110]. An enzymatic reaction using lactate oxidase (LOx), which generated an electrical signal according to the analyte concentration, was used to measure the level of lactate in sweat. This tattoo-like sensor was firmly attached to the skin and remained functional during repeated bending and stretching. Later, to enable non-invasive biofluid extraction and analysis, researchers developed a tattoo-based electrochemical sensor in combination with reverse iontophoresis [111].

A flexible multilayer biosensor patch for sweat glucose detection was designed by Lee et al. by utilizing a silicone-based substrate that maintained good skin conformity [112]. Through real-time calibration, the patch’s integrated glucose, pH, humidity, and temperature sensors enabled more accurate glucose measurement. Here, a sweat-absorbing layer enhanced sweat collection, while a waterproof layer reduced evaporation. The device also demonstrated excellent stability and reusability, emphasizing its potential for effective wearable glucose monitoring applications [113,114].

### 4.2. Saliva-Based Biosensing

Saliva provides a simple, non-invasive, and practical way to collect samples, resulting in diagnostic applications. Collecting samples from saliva is particularly advantageous for children, the elderly, and home-based health monitoring since, in contrast to many standard sampling techniques, it may be obtained with little discomfort and little training [115]. Saliva also contains a wide variety of biologically significant substances, including proteins, hormones, enzymes, and other indicators related to stress, the course of disease, and general health. These features have aided in the development of saliva-based diagnostic tools for the identification and tracking of several systemic and dental diseases, as well as mental health and stress-related disorders [116,117]. Despite these benefits, measurement accuracy may be impacted by issues including individual differences in biomarker concentrations, delays between changes in blood and saliva biomarker levels, and possible contamination. However, saliva-based diagnostics have enormous potential in transforming healthcare by enabling accessible, user-friendly, and continuous health monitoring [118].

Salivary biomarkers can have concentrations as low as femtomolar (fM) and may span to millimolar (mM) levels. The development of biosensors is more difficult because of this broad dynamic range, which is measured in about 10 orders of magnitude [119]. Khan et al. reported a paper-based electrochemical biosensor for the non-invasive detection of prostate-specific antigen (PSA) in saliva utilizing PSA-specific antibodies as the sensing component [120]. This method provides a promising alternative to conventional blood-based prostate cancer screening and treatment monitoring because salivary PSA levels have been demonstrated to correlate well with blood PSA concentrations. The sensor demonstrated an extensive linear detection range of 0.1 pg mL^−1^ to 100 ng mL^−1^, attaining a low detection limit of 40 fg mL^−1^. Its multilayer design, which included a graphene–polymer nanocomposite and filter paper, enhanced saliva absorption and helped achieve extremely sensitive PSA detection.

Salivary diagnostic procedures have been explored for the identification of infectious diseases. The COVID-19 pandemic encouraged research into wearable devices that can identify viral antigens, antibodies, and inflammatory indicators directly from saliva. A paper-based electrochemical biosensor for the detection of the SARS-CoV-2 spike protein receptor-binding domain (RBD) in saliva and swab specimens was reported in [121]. The sensor specifically recognized the target protein by employing the monoclonal antibody CR3022, which was made using a plant transient expression technique. The device demonstrated a wide linear response range of 0.1 pg mL^−1^ to 500 ng mL^−1^ and a low detection limit of 2.0 fg mL^−1^ using differential pulse voltammetry (DPV). As new variants of the SARS-CoV-2 virus continue to emerge, modifications to the recognition elements of biosensors are necessary to ensure accurate detection of the altered spike proteins. Electrochemical biosensors designed with variant-specific probes can address this challenge by enabling the simultaneous detection and discrimination of different spike protein variants [122].

Saliva-based analysis is a feasible non-invasive approach to therapeutic drug monitoring, enabling easier and more accessible home healthcare. It is especially beneficial for infants and the elderly, for whom intrusive collection of blood may pose difficulties or elevate the risk of infection. The drug concentrations in saliva frequently coincide with those in blood; the measurements taken from them can be used as substitutes for blood or plasma drug monitoring provided suitable correction factors are utilized [123]. Recent advances have focused on improving continuous saliva sampling through microfluidic mouthguards, flexible oral patches, and wireless intraoral sensing platforms. Nevertheless, saliva-based sensing remains challenging because biomarker concentrations are influenced by salivary flow rate, circadian rhythm, food intake, oral hygiene, and contamination. These factors necessitate calibration strategies to improve measurement reliability.

### 4.3. Tear-Based Biosensing

Tear fluid, sometimes referred to as the tear film, is a thin layer of biological fluid that covers and shields the eye’s surface. It includes a broad range of biological molecules and biomarkers, including glucose, lactate, electrolytes, proteins, cytokines, and hormones that are indicative of both ocular and systemic physiological states. Tear fluid has drawn a lot of attention as a useful source of diagnostic information for monitoring eye health as well as several systemic disorders due to its rich biochemical composition [124].

Wearable tear-based biosensors offer a non-invasive method of identifying analytes and biomarkers in tear fluid, enabling continuous real-time health monitoring. These biosensing systems are usually made utilizing well-established microelectromechanical system (MEMS) technologies, which include wet or dry etching, vacuum deposition, and photolithography [125]. Flexible and wearable sensing platforms can be developed by integrating sensing components onto soft substrates using printing, sputtering, and photolithography techniques. Furthermore, microfluidic structures utilized for the collection, transport, and analysis of tears are usually developed using soft lithography, micro-milling, 3D printing, laser micromachining, and injection moulding [126].

Smart contact lenses represent a highly innovative approach to tear biosensing, integrating flexible sensors, microelectronic components, wireless communication modules, and power management systems into soft contact lens materials. These devices enable continuous and non-invasive monitoring of tear biomarkers, offering significant potential for real-time health monitoring and personalized healthcare. For effective functioning of contact lens biosensors, the lens must fit appropriately on the surface of the eye and remain in close contact with the tear film. Between the contact lens and the corneal epithelium, there is a thin aqueous tear layer that supports the lens, and an outer lipid layer keeps the tear film stable and prevents evaporation. The precise collection and analysis of tear fluid are crucial for the reliable function of contact lens sensors. These devices may continually sample tear constituents to directly measure many biochemical and physiological parameters, such as glucose levels, pH, proteins, electrolytes, intraocular pressure, and corneal temperature, thereby providing non-invasive and real-time health monitoring [127].

Apart from contact lens biosensors, eyeglass-based wearable sensors have been developed as a non-invasive approach for real-time tear analysis [128,129]. Sempionatto et al. proposed an eyeglass-based wearable tear biosensor that integrates a microfluidic electrochemical sensing device into the nose pad of eyeglasses [130]. This device detects alcohol concentrations in tear fluid from immobilized alcohol oxidase, which converts ethanol into acetaldehyde and hydrogen peroxide. This design enables the non-invasive monitoring of tear biomarkers, thereby eliminating the potential risks of infection, discomfort, and vision obstruction associated with contact lens-based sensing systems.

Besides contact lens and eyeglass-based platforms, strip-based ocular sensors fabricated on rigid plastic substrates have also been investigated for tear analysis. These sensors have been used to monitor biomarkers and physiological parameters associated with conditions such as diabetes, keratoconjunctivitis sicca (dry eye disease), and tissue oxygenation through tear fluid analysis [131]. Despite their ability to detect clinically relevant indicators, strip-based sensors have several limitations. Their rigid structure can cause discomfort and irritation to the eye during use, and they generally lack integrated data processing and wireless communication capabilities. As a result, these sensors are less suitable for continuous wearable monitoring compared with newer flexible and smart tear-sensing platforms [132]. Continuous tear sensing is also affected by low tear secretion rates, evaporation, reflex tearing, and variations in tear composition. Moreover, the temporal delay between changes in blood glucose and tear glucose concentrations remains an important consideration for clinical interpretation. Future smart contact lenses incorporating real-time calibration algorithms and multiplexed sensing may improve measurement accuracy.

### 4.4. Interstitial Fluid (ISF) Based Biosensing

Interstitial fluid (ISF), which surrounds cells within the body’s tissues, has emerged as a promising biofluid for wearable sensing applications. ISF is a potential alternative to conventional blood sampling since it has many biomarkers that accurately represent blood composition. Unlike conventional blood sampling, ISF collection is generally less painful and avoids issues such as blood clot formation [133]. Recent advances in wearable ISF sensors have enabled continuous monitoring of a wide range of clinically relevant biomarkers, including glucose, lactate, electrolytes, hormones, proteins, cytokines, antibodies, and therapeutic drugs.

The size of the target biomarker is an important factor when selecting an appropriate ISF extraction technique. Some extraction methods, including suction blister formation, micropore-based sampling, and suction-assisted approaches, can cause minor tissue damage and trigger local inflammatory responses. Such effects may alter the concentrations of larger biomarkers, particularly proteins, leading to inaccuracies in measurement. To support minimally invasive health monitoring, several ISF extraction strategies have been successfully integrated with wearable biosensing platforms. Among the most widely used approaches are reverse iontophoresis, microneedle-based sampling (solid, hollow, porous, and hydrogel-based), and microdialysis, each enabling the collection and analysis of ISF for continuous biomarker monitoring while minimizing patient discomfort [134,135,136,137].

ISF contains a biomarker composition closely mirroring arterial blood, making it a prime candidate for continuous metabolic tracking. Microneedle (MN) arrays categorized into solid, hollow, porous, and hydrogel-swelling architectures penetrate the stratum corneum to access dermal ISF without stimulating nociceptors. Hydrogel-forming MNs uptake ISF via osmotic swelling, preserving analyte integrity, while hollow MNs integrated with microfluidic channels enable continuous amperometric sensing. Non-invasive ISF extraction frequently employs reverse iontophoresis (RI), applying a low-level electroosmotic current (≤0.5 mA/cm^2^) across the skin to drive charged and neutral molecules to the surface. However, sustained electric field exposure induces local skin irritation, transient pore formation (electroporation), cellular damage, and localized pH shifts at the electrode site. To mitigate RI-induced thermal and electrochemical skin damage, current protocols implement pulsed current profiles, biocompatible hydrogel buffers, and alternating polarity cycles. A fundamental limitation of biofluid sensing, particularly in ISF and sweat, is physiological detection latency relative to systemically circulating blood parameters. Biomarker transport from dermal capillaries to ISF or sweat glands is governed by molecular weight, capillary permeability, and local blood flow, inducing a time lag ranging from 5 to 20 min for glucose and lactate. Accounting for this transport delay is critical when applying biofluid metrics to acute clinical decisions [138].

Combined with electrochemical, optical, and other sophisticated sensing methods, these technologies deliver real-time physiological information, allowing for personalized healthcare. There are still issues with sensor stability, measurement accuracy, biofouling, biocompatibility, and motion-induced signal fluctuations despite tremendous advancements. Future advancements in flexible sensor architectures, artificial intelligence-assisted data processing, and anti-biofouling materials could improve the clinical usefulness and dependability of ISF-based wearable biosensors for point-of-care diagnostics and continuous health monitoring [139,140].

**Figure 4:**
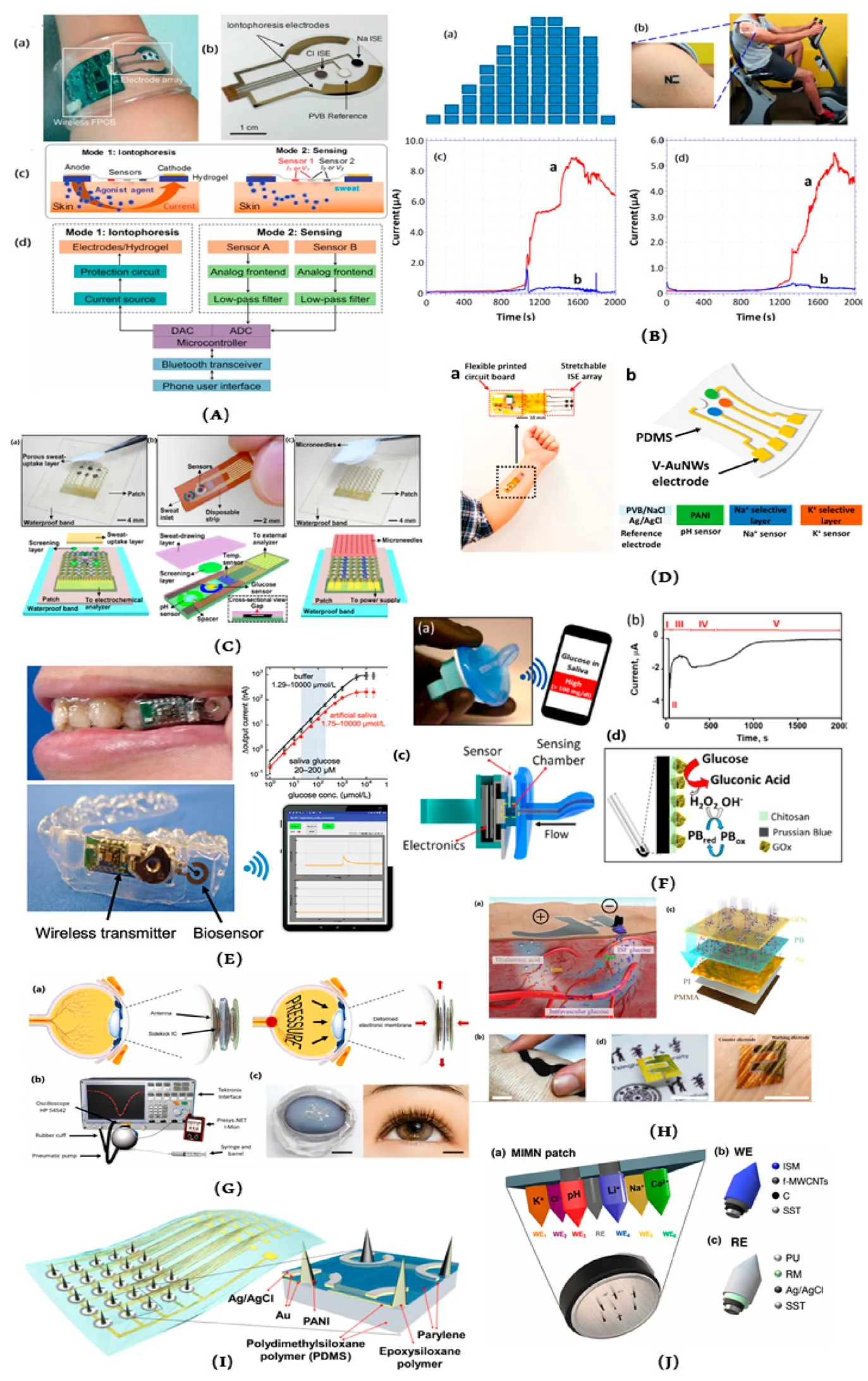
Examples of biochemical wearable sensors (**A**) Sweat sensor for the measurement of glucose, Na^+^, and Cl^−^ analysis. (**a**) Sweat extraction and sensing platform (**b**) iontophoresis and sweat sensor electrodes for Na^+^ and Cl^−^ sensing (**c**) schematic illustration of iontophoresis and sensing modes (**d**) System-level block diagram of the platform [107] (**B**) Monitoring of sweat lactate. (**a**) Exercise resistance profile on a stationary cycle. (**b**) An ‘NE’ lactate biosensor applied to a male volunteer (**c**,**d**) Response of Lox enzyme and enzyme-free biosensor using two representative subjects [110] (**C**) Wearable sweat monitoring device and microneedle-based transdermal drug delivery module. (**a**) Camera image (**top**) and schematic (**bottom**) of wearable sweat monitoring patch (**b**) Camera image (**top**) and schematic (**bottom**) of disposable sweat monitoring strip. (**c**) Camera image (**top**) and schematic (**bottom**) of transdermal drug delivery device [112] (**D**) Sweat analysis device. (**a**) Wearable device on a subject’s wrist. (**b**) Schematic of stretchable electrodes containing an Ag/AgCl reference electrode and pH, Na^+^, and K^+^ working electrodes [141]. (**E**) Photograph of the mouthguard biosensor integrated with wireless module and battery [142]. (**F**) Glucose pacifier sensor. (**a**) Real picture and use of pacifier biosensor (**b**) On-body saliva monitoring for a healthy individual. (**c**) Schematic of the assembled wireless pacifier biosensor. (**d**) Schematic of the glucose enzymatic biosensing approach of the electrode [143] (**G**) contact lens sensor for wireless monitoring of intraocular pressure. (**a**) Schematic showing the mechanism of intraocular pressure sensing. (**b**) Schematic of the experimental setup for wireless intraocular pressure sensing. (**c**) Photographs of the sensor transferred onto the contact lens worn by the eyeball [144] (**H**) Skin-like biosensor. (**a**) Schematic of electrochemical twin channel (ETC). (**b**) Flexible and biocompatible paper attached to the skin surface for ETC measurement. (**c**) Schematic of the ultrathin biosensor multilayers. (**d**) Selective electrochemically deposited dual electrode of the biosensor [145] (**I**) Flexible pH sensor array with epoxy siloxane polymer microneedle [146] (**J**) Microneedle-based sensor patch. (**a**) Multi-ion microneedle patch (**b**) Schematic of the working electrode layers. (**c**) Schematic of the reference electrode layers [147]. Reprinted with permission from corresponding references.

Although wearable biosensors monitor biomarkers from alternative biofluids, clinical diagnosis is generally based on blood biomarker concentrations. Consequently, numerous studies have investigated mathematical calibration and correlation models that relate biomarker concentrations measured in sweat, saliva, tears, and ISF to corresponding blood values. The accuracy of these models depends on physiological factors including sweat rate, tissue perfusion, hydration status, temperature, individual metabolism, and diffusion kinetics. Recent studies have therefore incorporated subject-specific calibration, machine learning algorithms, and multimodal sensor fusion to improve prediction accuracy and reduce inter-individual variability. Table 4 summarizes the common biochemical biomarkers in wearable biosensing. Figure 4 gives examples of wearable biochemical sensors proposed in the literature in recent years.

## 5. Integration of Biophysical and Biochemical Sensing

Although individual sensing modalities can provide significant physiological information, human health is impacted by numerous interrelated biological processes. Biophysical and biochemical processes may not occur independently, and so measurements acquired from a single sensing modality may not fully represent an individual’s health status [106,148]. To address this limitation, increasing attention has been directed toward multimodal wearable biosensors that can simultaneously monitor both biophysical and biochemical signals with increased sensitivity and mechanical flexibility [149,150]. The integration of these sensing modalities offers several significant benefits. It improves diagnosis reliability by integrating complementary physiological information gathered from several sources. Utilizing a variety of biomarkers also aids in lowering measurement uncertainty [109,151]. Additionally, the concurrent monitoring of biochemical and physical data offers important context for understanding physiological changes, resulting in better clinical decision-making and more accurate health evaluation.

The combination of biochemical sensors with physical sensors, including heart rate, SpO_2_, sweat rate, and temperature sensors, allows the acquisition of complementary physiological data from multiple sources. Such integrated sensing systems offer a more complete picture of human health and provide insights that may not be achievable through individual sensing modalities alone. As a result, multimodal monitoring can improve diagnostic accuracy and enable more effective health management. Using multiple sources of sensory information, these systems will help achieve a more comprehensive understanding of the state of an individual’s health and physical activity. This is because the combination of the sensor readings helps reduce uncertainties that may arise from single-parameter readings. In addition, auxiliary sensors such as the pH and temperature sensors may help calibrate enzymatic biosensors [152].

The concept of multimodal sensing has been demonstrated in recent investigations by integrating different sensing modalities into a single wireless platform. Zahed et al. developed a flexible patch-based wireless wearable system that integrates biophysical, biochemical sensing, and microfluidic technology in a single platform [153]. The device consists of a microfluidic glucose sensor using reduced graphene oxide and dry ECG electrodes for the monitoring of glucose levels in sweat and heart activity at once. To ensure high-precision measurements, the system also includes pH and temperature sensors for the calibration of glucose levels. The proposed device proved to be highly sensitive and stable, and its efficiency was confirmed by tests conducted on the human body during exercise. A wireless wrist-worn wearable system is introduced by Hong et al. that estimates post-exercise blood glucose levels from sweat glucose measurements [154]. The device integrated glucose and temperature sensors with optical sensors and an accelerometer to simultaneously monitor sweat glucose, heart rate, SpO_2_, and calorie expenditure. By combining these physiological and biochemical measurements with individual sweat-to-blood glucose correlation factors, the system was able to estimate blood glucose levels after exercise. Continuous multimodal monitoring also enabled the investigation of the relationship between physical activity and glucose regulation. Fang et al. created a novel epidermal patch that allows for real-time and simultaneous monitoring of biophysical and biochemical signals using a single platform [155]. This platform involved the integration of ultrasound sensors that allowed for the real-time monitoring of cardiovascular biophysical signals, namely heart rate and blood pressure, and electrochemical sensors that allowed for the detection of biomarker concentrations like lactate, caffeine, alcohol, and glucose in the sweat and interstitial fluids. The novel wearable device was tested in different physiological situations such as exercise, eating, alcohol intake, and caffeine intake.

Recent advances in wearable biosensing have led to the development of fully integrated systems with innovative architectures and self-powered functionalities aimed at reducing motion-related signal interference and enabling battery-free operation. One such platform was designed by Zhao et al. as a freestanding wearable device capable of simultaneously monitoring glucose, lactate, choline, and body movement through acceleration measurements [156]. The system incorporated an optional iontophoresis unit, a multilayer structure for biofluid handling, sensing and signal transduction components, and wireless data acquisition and transmission modules, with an overall power consumption of approximately 100 mW. Its distinctive out-of-plane structural design effectively reduced mechanical strain on the electrical connections, resulting in stable and noise-free amperometric measurements even during physical activity. The reliability of the device was evaluated by recording both biochemical and motion data while participants performed activities such as punching, arm swinging, and forearm twisting, demonstrating strong resistance to motion-induced artefacts. Yu et al. developed a biofuel-powered wearable biosensing system for monitoring urea, ammonia, glucose, pH, and skin temperature [157]. The device eliminated the need for conventional batteries by using a lactate-based biofuel cell to directly extract energy from perspiration. The platform successfully monitored several sweat biomarkers during physical activity and achieved self-powered operation by integrating sensing electronics, wireless communication modules, and signal processing circuits.

Ling et al. reported the development of an inkjet-printed stretchable wearable sensor patch for performing electrochemical sensing along with biopotential measurement simultaneously [158]. This system consists of a multi-layered structure with horseshoe-shaped serpentine structures, which makes it highly flexible and stable up to a strain of 20%. By proper surface functionalization, this sensor patch has been successfully developed for the continuous detection of potassium ions (K^+^) and glucose. Apart from biochemical sensing, the developed platform was also tested for recording biopotential signals. This shows the ability of the developed platform for physiological measurements. Hozumi et al. proposed a flexible multimodal wearable sensor platform that was able to measure glucose levels in sweat, ECG signals, and skin temperature [159]. This platform was specifically made to allow continuous measurement of biochemical and physiological parameters using skin-mounted sensors. For accurate glucose detection, a microfluidic channel was built into the platform to continuously refresh sweat at the sensing interface and to prevent analyte accumulation so that accurate measurements can be taken in real time.

**Figure 5:**
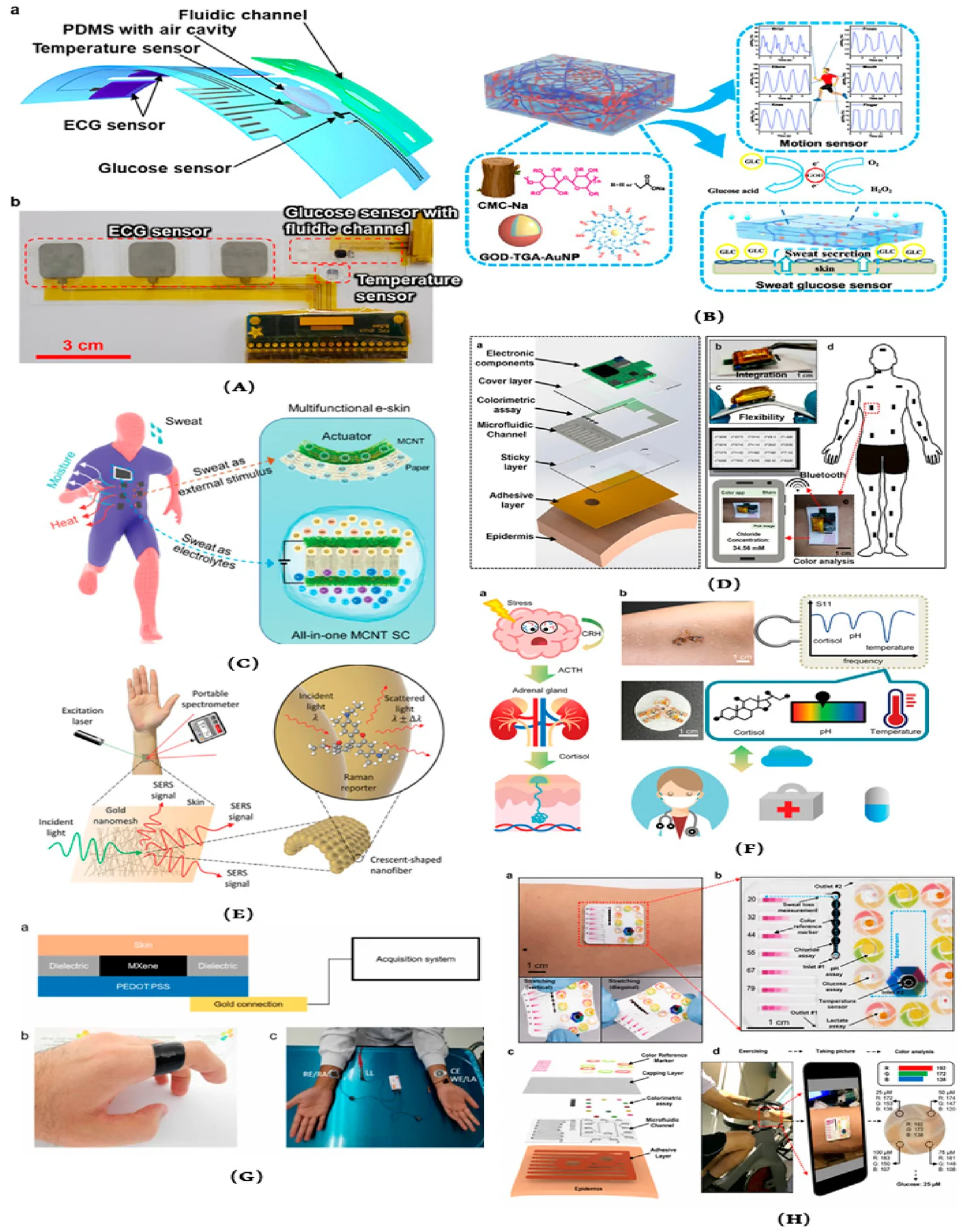
Examples of multimodal wearable sensors (**A**) proposed multimodal wearable sensors. (**a**) schematic (**b**) photograph of the wearable sensor sheet integrated with a sweat glucose sensor with a fluid channel, ECG sensor and skin temperature sensor [159] (**B**) Multifunctional hydrogel based sensor capable of simultaneous monitoring of motion and glucose [160] (**C**) Schematic representation of MCNT composite based electronic skin that combines energy storage and thermal management [161] (**D**) Schematic and photographs of wireless epidermal sensors used for monitoring body temperature and sweat. (**a**) Exploded view of sensing platform (**b**) Optical image of the system showing integration of a multifunctional sweat and temperature sensing platform. (**c**) Optical image of the complete system under bending condition. (**d**) Schematic of epidermal sensor. (**e**) Image of complete sensor attached to human skin during sweating [162] (**E**) Wearable SERS-based sensor for the sensitive detection of sweat biomarkers including glucose and electrolytes enabling non-invasive real-time monitoring of metabolic health during physical activity [167] (**F**) Multifunctional epidermal sensing platform for stress monitoring and health care. (**a**) Stress-triggered cortisol production and its transport to sweat through the hypothalamic–pituitary–adrenal axis. (**b**) Wearable resonance circuit-based sweat sensor for the concurrent monitoring of cortisol, pH, and temperature [168] (**G**) Skin-conformal printed MXene-based ECG sensing platform. (**a**) Schematic representation (**b**) Printed MXene ECG electrode patch attached to index finger. (**c**) Experimental configuration of EIS and ECG measurement [169] (**H**) Microfluidic wearable platform for long-term monitoring of biochemical and physiological parameters. (**a**) Flexible devices for on-skin colorimetric sweat analysis. (**b**) Top view illustration of blue-dyed microfluidic channel (**c**) Exploded schematic of the device structure and skin interface. (**d**) Schematic of sweat collection and digital image-based color analysis [170]. Reprinted with permission from corresponding references.

A multifunctional hydrogel-based sensor capable of simultaneously monitoring motion and glucose was developed by Zhou et al. by integrating piezoresistive and electrochemical sensing mechanisms [160]. Its layered nanocomposite architecture provides enhanced sensitivity while improving mechanical stability and durability. A multifunctional electronic skin based on an MXene carbon nanotube composite (MCNT) is developed by Wang et al. that combines energy storage with thermal management in a single wearable device [161]. Sweat functions as the electrolyte to power a supercapacitor for energy harvesting, while a moisture-responsive actuator enables adaptive heat dissipation. Owing to the superior conductivity and structural stability of the MCNT composite, the system maintains reliable performance even under extended exposure to perspiration. A multifunctional microfluidic wearable platform is proposed by Alam et al. that integrates colorimetric sweat sensing with wireless electronics to enable real-time monitoring of sweat chloride concentration and skin temperature [162]. By utilizing flexible microchannels for efficient sweat transport and a smartphone application for data visualization, the platform provides dynamic, noninvasive health assessment with enhanced user accessibility. Table 5 summarizes the comparison of recent wearable biosensing systems based on sensing modalities, materials, and clinical applications, and Figure 5 gives examples of multimodal wearable sensors proposed in the literature in recent years.

The rapid development of wearable biosensors that measure physiologically relevant parameters is generating huge volumes of data. These data streams include electrophysiological, biomechanical, biochemical, environmental, and behavioral signals recorded sequentially. To extract clinically relevant information from these complex and heterogeneous data, there is a need for advanced computing methods to identify patterns and relationships between several physiological variables. The use of artificial intelligence (AI), machine learning (ML), and deep learning (DL) technologies for wearable sensor data processing has become widely popular [163,164]. In addition to data analysis, AI significantly enhances the reliability and robustness of wearable biosensing systems. Although AI cannot directly repair sensor hardware, ML and DL algorithms can compensate for variations in sensor outputs resulting from bodily motions, ambient fluctuations, and long-term sensor drift. By identifying and resolving these sources of error, AI-based models enhance the accuracy, stability, and interpretation of real-time physiological measurements [165]. Edge AI architecture examines the data directly on wearable devices, eliminating the need to transmit information to remote cloud servers. This approach reduces latency, improves user privacy, and facilitates rapid decision-making. Additionally, edge AI is appropriate for continuous health monitoring under circumstances where computational power is constrained or communication networks have poor bandwidth. Edge AI enables real-time error correction and adaptive model updates, which help to keep the performance of wearables consistent in different conditions [166].

Multimodal wearable systems that combine different sensing functions have been developed to improve measurement accuracy and make the devices more reliable. These systems can combine information from multiple sensors, operate using self-generated power, and even predict biomarker levels after exercise. However, several challenges remain for long-term wearable monitoring. One of the main issues is the limited amount of power available from self-powered devices. Another challenge is reducing the energy required for sensor operation, sweat stimulation, and wireless data transmission. Therefore, careful power management is essential for the successful development of continuous wearable health monitoring systems.

**Table 5:** Comparison of Recent Wearable Biosensing Systems.

Sensing Modalities	Materials and Fabrication	Clinical Applications	Ref.
Strain deformations, Temperature, Sweat pH	Key materials include hydrogel and polydimethylsiloxane (PDMS). The sensor is a hydrogel-coated polydimethylsiloxane (PDMS) optical fiber (HPOF)	Real-time monitoring of heartbeat, Real-time monitoring of respiration, Real-time monitoring of body temperature, Real-time monitoring of sweat pH, Early disease prediction and diagnosis	[171]
Glucose, ECG, pH, Temperature	Flexible materials, reduced graphene oxide, MXene, polyvinylidene fluoride-based conductive carbon nanofiber. Fabrication Technique: Laser-burned.	Sweat glucose monitoring, ECG monitoring, and physiological monitoring	[153]
Glucose, potassium	Fabrication Technique: Inkjet printing. Multi-layer configuration with horseshoe-shaped serpentines.	Daily health monitoring, Real-time monitoring of K^+^, Real-time monitoring of glucose, Biopotential recording, On-body continuous biological quantification, Comprehensive healthcare management	[158]
Biaxial strain, Temperature, Humidity, Heart rate, SpO_2_ levels	NA	Emotion detection, Psychological health evaluation, Emotional support through telemedicine, Monitoring of biaxial strain, temperature, humidity, heart rate, and SpO_2_ levels	[172]
Temperature, sweat glucose, Sweat ammonium	A strain-resilient liquid metal composite and a soft substrate	In situ monitoring of Skin temperature during exercise, in situ monitoring of Sweat electrolytes during exercise, Prolonged monitoring of Sweat glucose in different subjects, Prolonged monitoring of Sweat ammonium in different subjects	[173]
ECG, Heart rate, Respiratory rate, Physical activities	Low-modulus, elastomeric system; stretchable nanomembrane sensors; hyperelastic membranes; thin-film stretchable skin electrodes.	Continuous ambulatory cardiac monitoring, Monitoring of ECG, heart rate, respiratory rate, and physical activities, Real-time detection of cardiac abnormalities, and multi-faceted clinically relevant analysis	[174]
Temperature, Humidity, Motion (via acceleration sensor)	Solution-based proportional integral (PI) as a flexible material. PET film as a topmost layer, Anisotropic conductive film (ACF) for bonding. Fabrication Techniques: Spin-coating for the PI layer on a rigid glass substrate. Lithographic process for patterning gold (Au) contact electrodes and serpentine-shaped interdigitated humidity sensor. Laser cutter technique for patterning the PET film.	Delivering real-time healthcare information to personalized smartphones. Assisting with emergencies due to “unpredictable” deviations in body information. Aiding in medical checkups for vulnerable patients. Early detection and continuous monitoring of temperature and sweat-related diseases. Sharp motion detection for universal healthcare settings. Detecting a fall. Monitoring human body trauma.	[175]
Electrolytes (sodium, potassium)—Metabolites (glucose, lactate)—Skin temperature	PET: mechanically flexible substrate for sensors. Ion-selective membranes are used for potentiometric electrolyte sensors. Ag/AgCl: likely for reference electrodes in the sensor array	Fitness and athletic monitoring, Remote health monitoring of toddlers, elderly people, and patients with disabilities or conditions requiring continuous caregiver attention, and in-situ analyses of other biomarkers within sweat and other fluid samples	[176]
Heart rate, Temperature, Strain/motion	PI as an insulating layer and for encapsulating. PDMS for adhesion, as a substrate, and as a matrix material for UV sensing. Fabrication techniques: Spin-casting	Monitoring of heart rate, Monitoring of temporal dynamics of arterial blood flow	[177]
SpO_2_, Heart rate, ECG, Seismocardiogram (SCG)	Fabrication method: cut-solder-paste microfabrication, rapid-prototyped via a dry, digital, and cost-effective freeform manufacturing process. Materials: Multilayer stack of stretchable layers.	Skin hydration, Skin temperature, Oxygen saturation level, Body motion, and continuous real-time blood pressure (BP) estimation	[178]
Strain/motion, Temperature, Electrophysiological (ECG, EMG)	Ag flakes/poly(3,4-ethylenedioxythiophene)-poly(styrenesulfonate) (Ag-PEDOT: PSS) composite. Fabrication Techniques: Stencil-based Fabrication	Health monitoring, Disease prevention, Electrophysiological signal monitoring (e.g., ECG, EMG), Accelerated healing of skin wounds, Cardiovascular diagnostics	[179]
Pressure, Temperature	Natural piezo-thermic transduction of human skin in conjunction with thin-film thermistors.	Wearable continuous blood pressure monitoring, Early diagnosis of cardiovascular disease	[180]
Glucose, Lactate, ECG, Temperature	Fabrication technique: screen-printing technology	Disease management, Health monitoring, Diabetes management (specifically, monitoring physiological effects of exercise in diabetic model rats, such as glucose decline), Exercise metabolism monitoring	[181]
Body temperature, Pulse rate, Sweat content	Thermoelectric hydrogel, poly (vinyl alcohol)/low acyl gellan gum, [Fe(CN)_6_]^4−/3−^ redox couple, glycerol, and Li^+^	Real-time human health assessment, Perception of body temperature, Perception of pulse rate, Perception of sweat content, Application in advanced intelligent medicine	[182]
Biopotential acquisition (ECG, EMG, EEG)	Fibrous thermoplastic polyurethane (TPU) for the scaffold. MXene-carbon for the strain sensing layer. MXene-waterborne polyurethane (MXene-WPU) for the electrode layer. Fabrication Technique: A scalable fabrication strategy is reported for the bioinspired E-skin (SPRABE-skin)	ECG, EMG, EEG monitoring, monitoring of running activities, and human-centred healthcare in daily life	[183]
Temperature, Skin Hydration	Nanofibrous soft substrates are used. Fabrication Techniques: Stamp-based transferring techniques are combined with electrospinning.	Fitness monitoring, Disease management, Measurement of bioelectrical signals, Measurement of temperature, Measurement of skin hydration, Measurement of many other physiological parameters	[184]
Glucose, lactic acid, uric acid, pH, Temperature	Bilayer unidirectional moisture transport nanomembrane. Fabrication Techniques: Thermal transfer manufacturing process.	Diabetes management (via glucose monitoring), Health monitoring (via glucose, lactic acid, uric acid, pH, temperature, and skin impedance monitoring), and real-time health monitoring	[185]

## 6. Conclusions and Future Perspectives

Flexible multimodal wearable biosensors have emerged as a very promising technology in modern healthcare, enabling a transition from post-disease treatment to proactive prevention. Over the past decade, significant progress in materials science, flexible electronics, microfabrication, nanotechnology, and wireless communication has substantially enhanced the efficiency of wearable sensing devices. These advancements led to the development of lightweight, skin-adherent, and biocompatible systems capable of continuously monitoring a wide range of physiological and biochemical information in real-world environments.

This review investigated current advancements in flexible multimodal wearable biosensors, starting with technological developments in materials and fabrication techniques. Flexible substrates, including PDMS, PI, PET, etc., enable the fabrication of comfortable wearable devices that adapt to body movements while ensuring consistent performance. The utilization of advanced nanomaterials, such as graphene, carbon nanotubes, MXenes, metallic nanowires, and conductive polymers, has enhanced sensor sensitivity, electrical conductivity, and mechanical durability. Fabrication methods like screen printing, inkjet printing, aerosol jet printing, laser-induced graphene synthesis, photolithography, and additive manufacturing have enhanced the advancement of scalable and configurable wearable sensing systems.

Biophysical sensing continues to play a key role in wearable health monitoring. Flexible sensors for measuring cardiovascular, respiratory, thermal, and motion-related parameters have shown significant potential in disease diagnosis, rehabilitation, and preventive healthcare. Additionally, biochemical sensing technologies have made significant progress. Biofluids, including sweat, saliva, tears, and interstitial fluid, provide access to significant biomarkers associated with metabolism, hydration, stress, inflammation, and disease progression. Improvements in microfluidics, nanostructured electrodes, molecular recognition elements, and microneedle-based systems have further enhanced sensing performance and reliability.

One of the main trends in recent times is the transition from single-parameter sensing to multimodal sensing. As the human body is complicated, the measurement of just one parameter may not give us all the necessary information. Wearable multimodal biosensors make use of biophysical and biochemical sensing techniques to obtain a holistic view of the person’s physiological state. The simultaneous sensing of electrophysiological signals, bodily motions, metabolic markers, and environmental conditions leads to improved accuracy of diagnosis and less ambiguity. AI has emerged as an integral part of multimodal biosensing wearable devices. There is a massive amount of data collected through these wearable devices, which needs advanced computational techniques for proper analysis. Machine learning, deep learning, and multimodal data fusion have led to significant advancements in predicting diseases, activity recognition, state of physiology, and personal health care analysis.

However, despite all these advancements, there are still some challenges that hinder the extensive usage of wearable biosensors in a clinical setting. It is very difficult to maintain the mechanical reliability of wearable devices since they need to work efficiently despite stretching and bending. There are more challenges like biocompatibility, biofouling, and the degradation of sensing elements that need to be considered. Another major challenge in the field is power consumption, especially when sensors continuously sense, transmit wirelessly, and process data. Although energy harvesting technologies such as triboelectric, piezoelectric, thermoelectric, and biofuel cell systems show considerable promise, fully self-powered operation remains an active area of research.

Data reliability and quality remain significant issues. Individual variations, environmental shifts, motion artefacts, and sensor drift can all have an impact on measurement accuracy and lower clinical trust in the information collected. Future wearable systems will necessitate adaptive calibration approaches, individualized analytical models, and sophisticated signal processing techniques to guarantee long-term monitoring. Another key challenge is the commercial development of lab prototypes into approved clinical devices that are commercially viable. While a significant number of wearable biosensors have been published in scientific literature, not many have managed to make it to regular clinical use. Large clinical trials, standard procedures for testing, regulatory processes, and cooperation between various stakeholders such as scientists, clinicians, industry partners, and regulatory agencies will all be needed to accelerate commercialization and clinical adoption.

## Figures and Tables

**Table 1:** Comparison of common flexible substrates for wearable sensors.

Substrate	Elasticity	Modulus (Approx.)	Key Advantages	Limitations	Typical Applications
PDMS	>100% strain	~750 kPa	Elastic, transparent, biocompatible, gas permeable	Hydrophobic, requires surface treatment	Sweat sensors, microfluidics, optical sensors
TPU	50–200% strain	~10–50 MPa	Abrasion resistant, roll-to-roll compatible, robust	Lower elasticity than silicones	Smart textiles, flexible electrodes
Ecoflex	>900% strain	~60 kPa (skin-like)	Ultra-soft, stretchable, comfortable	Low tear resistance, UV degradation	Epidermal patches, wearable physiological monitors
PI	<5% strain	~2–3 GPa	High thermal stability, thin-film compatible	Limited stretchability	Hybrid circuits, implantable sensors
PET	<5% strain	~2–4 GPa	Transparent, dimensionally stable, low-cost	Brittle under strain	Flexible displays, thin-film biosensors

**Table 2:** Comparison of sensing performance of flexible sensing materials.

Material	Target Analyte	Sensing Mechanism	Sensitivity	Detection Limit (LOD)/Linear Range	Signal Stability/Drift	Sweat Interference Resistance
MXenes (Ti_3_C_2_T_x_)	Lactic acid	Enzymatic/Amperometric	18.5 µA mM^−1^ cm^−2^	LOD: 12 µM; Linear range: 0.05–15 mM	Low initial drift; subject to oxidation in aerobic biofluids over >48 h	High hydrophilicity reduces non-specific protein adsorption; low cross-reactivity with uric acid.
MXenes (Ti_3_C_2_T_x_)	Glucose	Enzymatic (GOx)	42.1 µA mM^−1^ cm^−2^	LOD: 5.4 µM; Linear range: 0.01–10 mM	Metallic conductivity preserves signal baseline under repeated flexure	Excellent hydrophilic surface; susceptible to ascorbic acid interference without protective membranes
Carbon Nanotubes (CNTs/MWCNTs)	Lactic acid	Enzymatic (LOx)/Mediator-based	12.3 µA mM^−1^ cm^−2^	LOD: 25 µM; Linear range: 0.1–20 mM	Moderate drift due to biofouling; stable baseline over >1000 strain cycles	Hydrophobic surface prone to non-specific protein adsorption; chitosan or zwitterionic coatings improve resistance
Carbon Nanotubes (CNTs/MWCNTs)	Glucose	Enzymatic/Nanocomposite	28.6 µA mM^−1^ cm^−2^	LOD: 10 µM; Linear range: 0.05–12 mM	Highly stable electromechanical baseline (Gauge Factor ≈ 10–100)	Susceptible to Na^+^/K^+^ interference from sweat; mitigated using ion-selective membranes
PEDOT:PSS	Lactic acid	Organic Electrochemical Transistor (OECT)	112 µA mM^−1^ (transconductance)	LOD: 2.1 µM; Linear range: 0.01–5 mM	High transconductance amplification; hydration-induced signal drift over time	High swelling in sweat may alter impedance; crosslinking (e.g., GOPS) improves stability
PEDOT:PSS	Lactic acid/Glucose	Amperometric/Conductive Polymer	15.8 µA mM^−1^ cm^−2^	LOD: 8.0 µM; Linear range: 0.02–8 mM	Moderate drift under ambient humidity variations; excellent mechanical flexibility	Permselective membranes (e.g., Nafion) block anionic sweat interferents such as urate and acetaminophen.

**Table 3:** Biosensing physical modalities in wearable health monitoring.

Sensing Modality	Measured Parameter	Common Materials	Major Applications
ECG	Cardiac electrical activity	Graphene, Ag nanowires, hydrogels	Arrhythmia detection, cardiovascular monitoring
PPG	Blood volume changes	Flexible photodetectors, LEDs	Heart rate, SpO_2_, blood pressure
EMG	Muscle electrical activity	CNTs, MXenes, conductive polymers	Rehabilitation, prosthetic control
EEG	Brain electrical activity	Hydrogels, graphene electrodes	Sleep monitoring, neurological assessment
Temperature	Skin/body temperature	MXenes, CNTs, thermoelectric materials	Fever detection, metabolic monitoring
Respiratory Sensors	Breathing patterns	PVDF, graphene, CNTs	Sleep apnea, pulmonary assessment
Motion Sensors	Body movement	Strain sensors, IMUs	Human activity recognition, rehabilitation

**Table 4:** Biochemical biomarkers in wearable biosensing.

Biomarker	Biofluid	Clinical Significance
Glucose	Sweat, ISF, Tears	Diabetes management
Lactate	Sweat, ISF	Exercise physiology, tissue oxygenation
Sodium	Sweat	Hydration assessment
Potassium	Sweat	Electrolyte balance
Cortisol	Sweat, Saliva	Stress monitoring
Uric Acid	Sweat, ISF	Gout, metabolic disorders
Alcohol	ISF, Sweat	Alcohol consumption assessment
Therapeutic Drugs	ISF	Personalized medicine

## Data Availability

No new data were created or analyzed in this study. Data sharing is not applicable to this article.
